# Air quality modeling in the metropolitan area of São Paulo, Brazil: A review

**DOI:** 10.1016/j.atmosenv.2023.120301

**Published:** 2024-02-15

**Authors:** Mario Gavidia-Calderón, Daniel Schuch, Angel Vara-Vela, Rita Inoue, Edmilson D. Freitas, Taciana Toledo de A. Albuquerque, Yang Zhang, Maria de Fatima Andrade, Michelle L. Bell

**Affiliations:** a Departamento de Ciências Atmosféricas, Instituto de Astronomia, Geofísica e Ciências Atmosféricas, Universidade de São Paulo, 05508-090, São Paulo, Brazil; b Department of Civil and Environmental Engineering, Northeastern University, Boston, MA 02115, USA; c Department of Geoscience, Aarhus University, 8000 Aarhus, Denmark; d Department of Physics and Astronomy, Aarhus University, 8000 Aarhus, Denmark; e Departamento de Engenharia Sanitária e Ambiental, Universidade Federal de Minas Gerais, 31270-901, Belo Horizonte, Brazil; f School of Forestry & Environmental Studies, Yale University, New Haven, CT 06511, USA

**Keywords:** Air quality models, Ozone, PM_2.5_, Model performance evaluation, São paulo

## Abstract

Numerous studies have used air quality models to estimate pollutant concentrations in the Metropolitan Area of São Paulo (MASP) by using different inputs and assumptions. Our objectives are to summarize these studies, compare their performance, configurations, and inputs, and recommend areas of further research. We examined 29 air quality modeling studies that focused on ozone (O_3_) and fine particulate matter (PM_2.5_) performed over the MASP, published from 2001 to 2023. The California Institute of Technology airshed model (CIT) was the most used offline model, while the Weather Research and Forecasting model coupled with Chemistry (WRF-Chem) was the most used online model. Because the main source of air pollution in the MASP is the vehicular fleet, it is commonly used as the only anthropogenic input emissions. Simulation periods were typically the end of winter and during spring, seasons with higher O_3_ and PM_2.5_ concentrations. Model performance for hourly ozone is good with half of the studies with Pearson correlation above 0.6 and root mean square error (RMSE) ranging from 7.7 to 27.1 ppb. Fewer studies modeled PM_2.5_ and their performance is not as good as ozone estimates. Lack of information on emission sources, pollutant measurements, and urban meteorology parameters is the main limitation to perform air quality modeling. Nevertheless, researchers have used measurement campaign data to update emission factors, estimate temporal emission profiles, and estimate volatile organic compounds (VOCs) and aerosol speciation. They also tested different emission spatial disaggregation approaches and transitioned to global meteorological reanalysis with a higher spatial resolution. Areas of research to explore are further evaluation of models’ physics and chemical configurations, the impact of climate change on air quality, the use of satellite data, data assimilation techniques, and using model results in health impact studies. This work provides an overview of advancements in air quality modeling within the MASP and offers practical approaches for modeling air quality in other South American cities with limited data, particularly those heavily impacted by vehicle emissions.

## 1 Introduction

The Metropolitan Area of São Paulo (MASP) is the largest megacity in South America and it is commonly positioned in the top ten most populated cities in the world (United Nations, 2018). Like many other megacities, the MASP suffers from high levels of air pollution, being ozone (O_3_) and fine particulate matter (PM_2.5_) the pollutants that frequently exceed the state air quality standard. To quantify the levels of air pollution in the State of São Paulo, the State Environmental Agency (CETESB) deployed an air quality network to measure criteria pollutants, becoming one of the air quality networks with most spatial coverage in South America (Riojas-Rodríguez et al., 2016).

Air quality networks are insufficient to fully characterize the air quality of a region. They are expensive for installation and maintenance, which can be a limitation, especially in developing countries. Because an air quality station only produces information for one point in space, it is difficult to know all the conditions that led to the measured concentrations (Zhong et al., 2016). Even with one of the best air quality networks, monitoring data is not available for all time periods and locations of interest in the MASP. For example, air quality stations are mainly located inside the City of São Paulo, they are mainly located in urbanized areas, and not all the stations measure the same pollutants.

Air quality modeling is another approach to estimate the pollutant concentrations. They are a mathematical computer code that represents the physics, dynamics, radiative, and chemical processes of the atmosphere (Jacobson, 2005). But air quality models have uncertainties based on the quality of their inputs (e.g. the emission inventory, land-use data, elevation, etc), and the limited knowledge we have to describe a phenomenon (e.g. turbulence, precipitation, urban physics, etc). Therefore, they require evaluation by the government and the scientific community before they can be used to address research questions that are difficult to answer with monitors: What are the concentrations of species that are not measured by the air quality network? What physical-chemical formation processes lead to that measurement? And how different emission scenarios or meteorological conditions will affect the concentrations? (Simon et al., 2012).

In the MASP, several studies have used air quality models to estimate pollutant concentrations to answer different research questions. In this review, we covered the studies that used Eulerian 3-D air quality models, performed in the MASP that focused on O_3_ and PM_2.5_, published between 2001 and 2023. Our goals, therefore, are to summarize their configuration and inputs, compare their performance, and recommend areas of further research based on the limitations we found. This work is of interest to the air quality modeling community in South America, as it is the first review focused on a South American city. The experience in the MASP can help to perform air quality modeling in cities of the continent where the data is scarce and the vehicular fleet is the main source of pollution.

We start by describing the characteristics of the MASP air pollution. Then we summarize existing air quality model studies for the MASP. We describe their input emissions, their configurations (chemical and meteorological boundary conditions, chemical mechanism, aerosol modules, photolysis schemes, domain configurations, and physics options), their simulation performance based on the comparison with observations, and the scientific questions they answered. We end the review by identifying limitations and recommendations for future air quality studies for the MASP.

### 2 Air pollution in the metropolitan area of São Paulo

**Fig. 1:**
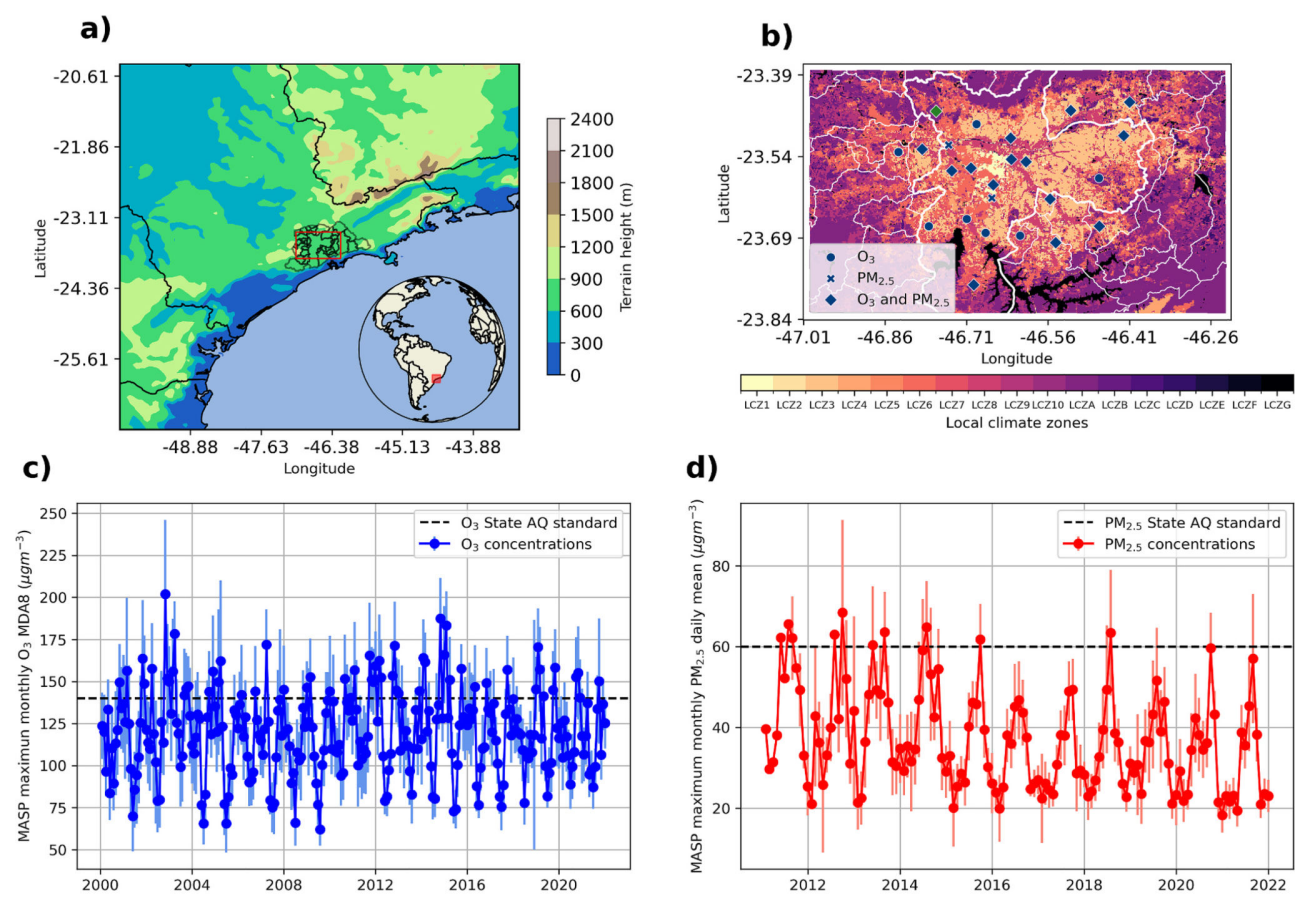
Key features of air quality in the MASP. A) Location and topography of the MASP, b) Local climate zones (LCZ) in the MASP (Stewart and Oke, 2012), and location of air quality stations: dots denote O_3_ measurements, exes denote PM_2.5_ measurements, and diamonds denote both pollutant measurements. The green diamond shows the location of Pico do Jaraguá station. The city of São Paulo is highlighted in thick white line c) Mean maximum monthly MDA8 Ozone from available air quality station in the MASP (The dashed line is the Air quality standard for O_3_ = 140 μg m^−3^ 8 h rolling mean, vertical lines show the standard deviation) and d) Monthly Maximum PM_2.5_ daily averages from available air quality station in the MASP (The dashed line is the air quality standard for PM_2.5_ = 60 μg m^−3^ daily average, the vertical lines show the standard deviation). Data in c) and d) come from the automatic air quality stations of CETESB.

The MASP is located in the State of São Paulo in Southeast Brazil (Fig. 1a). It has an area of 8000 km^2^ and it is populated by 21.9 million people (IBGE, 2020). By its location, the MASP presents a subtropical climate (Andrade et al., 2017). The South Atlantic Convergence Zone (SACZ) is one of the main atmospheric systems affecting precipitation during the summer, while the polar and subpolar jet streams affect the formation of fronts that reach the MASP. Air masses from the south pole produce cold fronts, enhancing thermal inversions and winds from Southeast to Northwest. During the pre-frontal systems, wind direction changes to Northwest and then to Southeast (Andrade et al., 2004).

As the MASP is close to the littoral (~60 km to the coast, see Fig. 1a) the sea breeze is an important factor in pollutant dispersion. Freitas et al. (2007) showed that during winter the MASP urban heat island accelerates the sea breeze up to the city center, where the sea breeze is delayed. This means that urban characteristics of MASP already affect its urban climate. This is aggravated by the unorganized development of the city, which has created an even more heterogeneous urban morphology (Lima and Maganñ Rueda, 2018) (Fig. 1b).

In the MASP, O_3_ and PM_2.5_ concentrations frequently exceed the São Paulo State air quality standards (140 μg m^−3^ or ~ 70 ppb 8-h rolling mean for O_3_ and 60 μg m^−3^ daily average for PM_2.5_) (CETESB, 2021). Fig. 1c shows that the maximum monthly MDA8 O_3_ frequently exceeds the state standard. Fig. 1d shows that the maximum monthly PM_2.5_ daily means have passed and are very close to the PM_2.5_ state air quality standard. Both figures highlight that even with the implementation of emission control policies, O_3_ and PM_2.5_ concentrations have not been reduced (Carvalho et al., 2015; Pérez-Martínez et al., 2015).

Ozone concentrations are frequently higher in spring (September to November). During summer (December to February) high concentrations are also measured but depend on the meteorology conditions (i.e. no precipitation) (Carvalho et al., 2015; Schuch et al., 2019). For PM_2.5_ spring is also the season when higher concentrations are observed, because September and October are periods when biomass burning takes place. Winter (June to August) is the season with the highest levels of primary pollutant concentrations. In winter, the dry period, high-pressure systems (blocking highs) produce clear skies, radiative inversions, and low wind speeds that increase the concentration of primary pollutants (Carvalho et al., 2015; Ulke and Andrade, 2001).

The vehicular fleet is the primary source of precursors and direct emission of regulated air pollutants in the MASP (Andrade et al., 2017; CETESB, 2021). The vehicular fleet is characterized by the extensive use of biofuels, which creates a particular atmosphere with high O_3_-forming volatile organic compounds (VOCs) (Alvim et al., 2020). According to CETESB (2021), from the total emissions, the vehicular fleet is responsible for 96% of carbon monoxide (CO), 73% of VOCs, 65% of nitrogen oxides (NO_X_), 40% of particulate matter (PM), and 11% of sulfur oxides (SO_X_) emissions. It extensively uses gasohol (a mixture of 78% gasoline and 22% ethanol) and biodiesel (diesel with 8–10% biodiesel). Half of the low-duty vehicular fleet is flex-fuel, which can run with any amount of gasohol and ethanol (CETESB, 2021). Emission strategies were implemented to reduce air pollution, including the Air Pollution Control Program for Motor Vehicles (PROCONVE) and the Air Pollution Control Program for Motorcycles and Similar Vehicles (PROMOT), resulting in a reduction of primary pollutants even with the increase of the vehicular fleet (Andrade et al., 2017).

The MASP is a VOC-limited atmosphere due to the high levels of NO_X_ emitted by the diesel heavy-duty fleet (Sánchez-Ccoyllo et al., 2006). But recent studies performed during the COVID lockdown found that this situation is not homogeneous for all urban areas. Sokhi et al. (2021) illustrated that in the same urban area, there are NO_X_-limited and VOC-limited controlled regions. The same was shown for other South American cities by Seguel et al. (2022) in an analysis also during the COVID lockdown, for São Paulo, Santiago, Lima, and Bogota. All these cities presented different behavior concerning O_3_ and PM_2.5_ concentrations, not only due to emission sources but also due to the meteorological conditions and topography. Consequently, air quality modeling need to represent the meteorological conditions over the MASP and correctly calculate and distribute its emissions inventory to estimate pollutant concentrations.

## 3 Air quality models used in the MASP

To the best of our knowledge, Bischoff-Gaub et al. (1998) performed the first air quality modeling in the State of São Paulo. It was not performed in the MASP but in Cubatão, an industrial area located closer to the State of São Paulo coast. The authors simulated SO_2_ concentrations using a modeling system that includes the Karlsruher Atmospheric Mesoscale Model (KAMM) and *Dreidimensionales Ausbreitungs-und Immissions-Simulationsmodell* (DRAIS) dispersion model (Adrian and Fiedler, 1991). The following year, as far as we know, the first work using neural network models to estimate ozone formation in the MASP was published by Guardani et al. (1999). We found that the use of Eulerian 3-D air quality models started at the beginning of the 2000s with the work of Ulke and Andrade (2001).

**Fig. 2:**
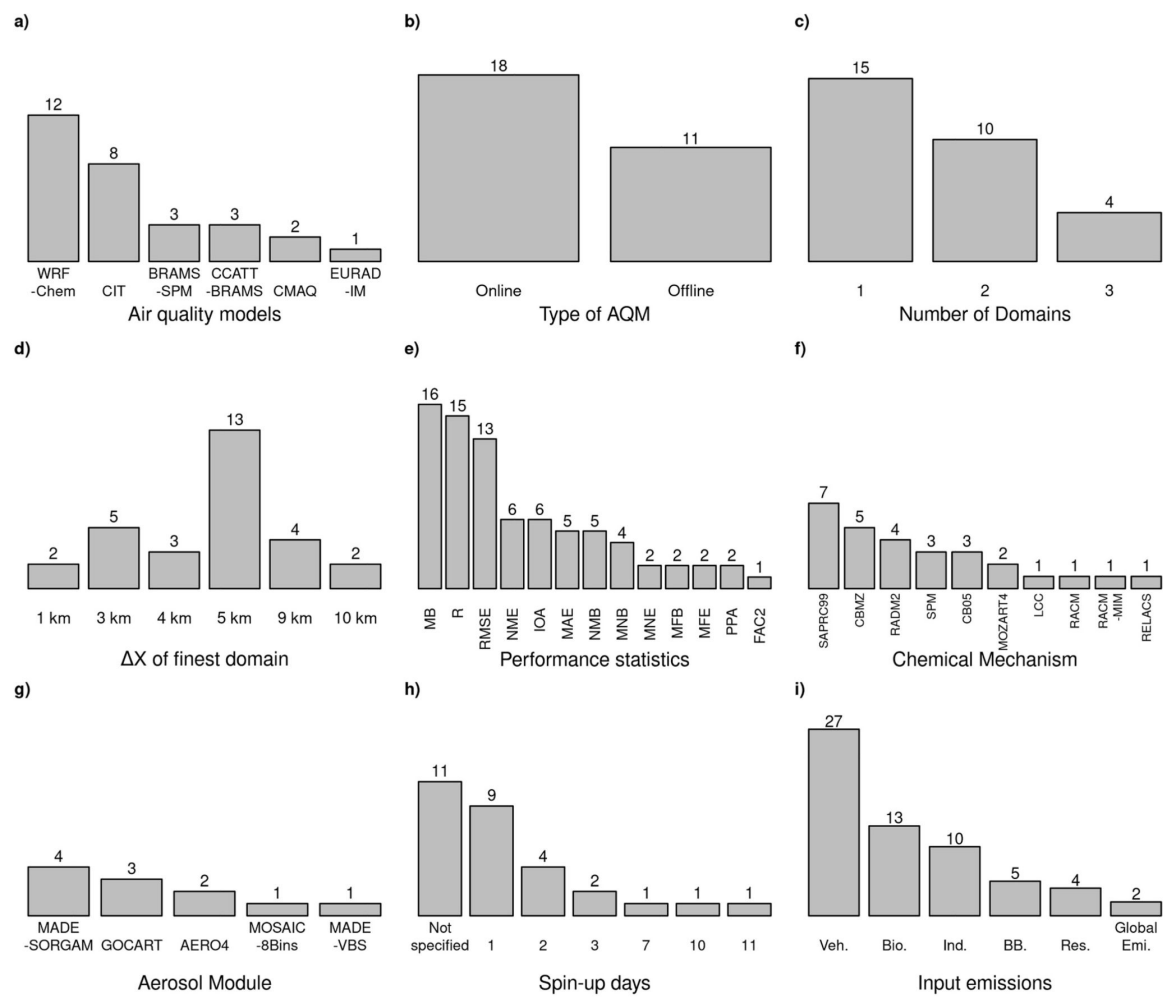
Frequency of different configuration features in our studies sample. In e) Statistics, MB: Mean bias, R: Pearson correlation, RMSE: Root mean square error, IOA: index of agreement, MAE: Mean absolute error, NME: Normalized mean error, MNB: Mean Normalized bias, MFB: Mean Fractional bias, NMB: Normalized mean bias, MNE: Mean Normalized Error, MFE: Mean fractional error, PPA: Pair peak accuracy, FAC2: Fraction of prediction within a factor of two. In i) Input emissions, Veh.: Vehicular, Ind: industrial emissions, Bio: Biogenic emissions, Res: Residential emissions, BB: Biomass burning emissions, Global Emi.: Total anthropogenic emissions from global emission inventories.

We carried out a systematic literature review regarding air quality modeling in the MASP. Our dataset consists of 29 modeling studies with Eulerian 3-D air quality models performed over the MASP or that included it inside their simulation domains. These studies covered a period of 23 years from 2001 to 2023. We selected forecast and post-analysis simulation studies. Table 1 shows the selected studies for this review. In the Supplementary Material, we present a brief description of the six air quality models used in the MASP and shown in Table 1 (Fig. 2a).

Our dataset has 27 studies (93 %) that performed post-analysis simulations. It also has 16 studies using offline models (55 %) and 13 using online models (45 %). Offline models require meteorological predictions generated separately to simulate the pollutant concentrations. The meteorological prediction usually comes from a meteorological simulation that could have different spatial and temporal resolutions; therefore, interpolation is required. Online models, on the other hand, calculate the meteorological fields and pollutant concentrations within one model system using the same grid and time-step of integration. The major difference between both types of models is that online models can address the feedback between the meteorological and chemistry components of the atmosphere (e.g. aerosol feedback to photolysis and radiation via direct effects and to cloud and precipitation via indirect effect) (Baklanov et al., 2014; Zhang, 2008; Zhang et al., 2012a, 2012b).

Although in some cases, results from other pollutants were included as they helped to explain the model results (i.e. NO_X_ and CO), we mainly focused on O_3_ and PM_2.5_. They are pollutants with a higher number of air quality standard violations, with important health impacts and climate implications.

## 4 Emissions used in air quality models

All air quality models in Table 1 require an emission inventory to run. An emission inventory describes the mass of pollutants released to the atmosphere by source for a given time and space (Pulles and Heslinga, 2007; Vallero, 2014). Pulles and Heslinga (2007) stressed that its estimation is a difficult task, and it is usually pointed as the main cause of differences between model results and observations.

Processing the emissions into the model is demanding. Besides knowing the total emissions, they need to be distributed in space and in time, and to be speciated according to the selected chemical mechanism or aerosol module (Matthias et al., 2018). In the MASP, from the local emission inventory developed by CETESB, researchers speciated NO_X_ (into NO and NO_2_), and the total VOCs emissions into the different organic species. PM emissions are also speciated into fine and coarse particle emissions, and in their components like SO_4_, NO_3_, organic carbon (OC) and elemental carbon (EC). Laboratory and field experiments have helped in this speciation endeavor. The same speciation is required when global emission inventories are used.

Some modeling systems have an emission preprocessor to assimilate the local emission inventory, but they are difficult to use as they require much detailed information that is limited in South American cities. For example, The Sparse Matrix Operator Kernel Emissions (SMOKE) modeling system or the *emiss_v03* tool, for CMAQ or WRF-Chem respectively, represent a real challenge to implement and usually many assumptions are made to use them. There are also emission pre-processors that work for different models like PREP-CHEM-SRC that create emission for WRF-Chem and CCATT-BRAMS models from global emission inventories (Freitas et al., 2011). In most cases, researchers developed their own emissions preprocessors or emission files for ad-hoc simulations (Andrade et al., 2015; Vara-Vela et al., 2016; Gavidia-Calderón et al., 2018; Ibarra-Espinosa et al., 2018; Schuch et al., 2020). For that reason, the studies in our dataset have used different emission estimates, calculated using different methodologies and distributed in space and time with different proxies.

In the MASP air quality simulations, the most used emission sources are anthropogenic emissions, biomass burning emissions, and biogenic emissions. As the MASP has more detailed information, it is common to extrapolate the emission information from the MASP to other cities located inside the simulation domain (Andrade et al., 2015).

### 4.1 Anthropogenic emissions

Because the vehicular fleet is the main source of air pollution in the MASP, 17 (58.6 %) studies only used vehicular emissions to account for anthropogenic emissions. This approximation usually works for the representation of O_3_ but is incomplete to estimate PM_2.5_ as part of its emission sources are not yet quantified (e.g. industry, road resuspension, etc.).

To calculate the vehicular emissions, researchers used the emissions factor and intensity use values from CETESB’s air quality reports (https://cetesb.sp.gov.br/ar/publicacoes-relatorios/), vehicular emissions reports (https://cetesb.sp.gov.br/veicular/relatorios-e-publicacoes/), and from tunnel experiments (Martins et al., 2006b; Nogueira et al., 2014; Pérez-Martínez et al., 2014). Emission factors from CETESB reports have been mostly combined with those obtained from tunnel experiments, as they more closely reflect the real-drive conditions in the MASP. There are emission factors for each type of vehicle (e.g. heavy-duty vehicles, light duty-vehicles, motorbikes, etc.) and for each type of fuel (e.g. ethanol, gasohol, and diesel). Besides considering exhaust emissions, evaporative emissions are also included as they are an important source of VOCs (Andrade et al., 2017). One of the challenges is accounting for flex-fuel vehicle emissions because they can operate rather with ethanol or gasoline which usually depends on the fuel prices (Salvo and Geiger, 2014).

The hourly temporal distribution for vehicular emissions is performed by assuming double Gaussian distributions to represent morning and late afternoon rush hours (Guerrero et al., 2021). Recent works used vehicular count profiles for light and heavy-duty vehicles from tunnel experiments as shown in Martins et al. (2006a) and Andrade et al. (2015). Nevertheless, most of the studies did not consider weekday variation; instead, a standard day emission along the simulation period is used. This situation has implications for representing the high ozone weekend effect that happens in the MASP (Andrade et al., 2017).

The spatial distribution is based on different proxies. For example, total vehicular emissions are distributed based on the street length in each grid cell using the approach of Andrade et al. (2015), on nocturnal lights satellite images (Gavidia-Calderón et al., 2018; Albuquerque et al., 2018), or on different ratios to distribute emission between urban and industrial land use types (Freitas et al., 2007). Other approaches include the distribution of emission inventories based on population density and total vehicular fleet (Andreão et al., 2020). Finally, Martins and Andrade (2008b) and Silva Junior and Andrade (2013) used a CO emissions map calculated using a traffic simulation from EMME/2 software for the MASP as a spatial proxy.

New emission preprocessors such as the VEIN emission model (Ibarra-Espinosa et al., 2018) improves the representation of the vehicular emissions in the MASP. It includes, besides the exhaust and evaporative emissions, emissions from the cold-start process. It is also able to compute the emission profile based on GPS count (Ibarra-Espinosa et al., 2020), which include weekday variation.

Global emissions inventories are often used to include other anthropogenic emissions sources. The Emissions Database for Global Atmospheric Research (EDGAR) and the Global emission data set developed with the GAINS model have been used in the MASP. Hoshyaripour et al. (2016), Vara-Vela et al. (2018), and Peralta et al. (2023) used local vehicular emission inventories together with EDGAR-HTAP global emissions to include industrial, domestic, and shipping emissions. Current versions of global emissions inventories (e.g. EDGAR6) have a spatial resolution of 0.1° which is suitable for simulation at 9 km, but still have limited spatial representation for simulations at higher resolutions. Furthermore, as shown in Huneeus et al. (2020) there are bigger differences between global emissions inventories for South America in sector aggrupation of each global emission inventory. Uncertainties of emissions for Brazil from EDGAR 4.3.2 are high, for example, 44.7 % for SO_2_, 123.5 % for NO_X_, 123.4 % for CO, 146.5 % for non-methane VOC (NMVOC), and 56.5 % for PM_2.5_ (Crippa et al., 2018).

Local information is limited about other anthropogenic sources other than ground transport, like energy, industries, domestic, ship, and aviation emissions. CETESB, besides including vehicular emissions, also published industrial total emissions without their location or information about their temporal variation, which limits its spatial and temporal distribution. More detailed information about vehicular emissions and the lack of information from other sources is another reason for its common use in air quality modeling in the MASP as the only anthropogenic emission source.

### 4.2 Biomass burning emissions

Biomass burning episodes are common in South America during the dry season, between August and October (Hoelzemann et al., 2009). Aerosols from these biomass-burning episodes in the Amazon can be effectively transported to urban areas in southeastern South America, such as the MASP (Vara-Vela et al., 2021). In addition, high concentrations of particulate matter in the MASP during the dry season have been attributed to the transport of aerosols from areas affected by sugarcane burning in inland regions (Pereira et al., 2017). Because biomass burning emits elemental carbon, organic carbon, and PM_2.5_, it is important in simulating particulate matter concentrations. Biomass burning is also important in the simulation of O_3_, as gas species such as CO, SO_2_, and VOCs are also emitted.

In studies using WRF-Chem, Hoshyaripour et al. (2016), Vara-Vela et al. (2018), and Benavente et al. (2023) incorporated biomass burning emissions using the Fire Inventory from NCAR (FINN) emission model. In the case of CCATT-BRAMS studies, Longo et al. (2013) employed the Brazilian Biomass Burning Emission Model (3BEM). Both FINN and 3BEM models provide daily emissions from open biomass burning, including wildfires, agricultural fires, and prescribed burning, on a global basis and at a resolution of 1 km^2^. Recent approaches that couple these models with fire radiative power (FRP) observations have shown significant improvements in representing particulate matter (Pimonsree et al., 2018; Kumar et al., 2022). Therefore, the utilization of FRP-based tools could result in an overall enhancement of air quality simulations over MASP, particularly during severe long-range transport events.

### 4.3 Natural emissions

In works using WRF-Chem, Vara-Vela et al. (2016), Gavidia-Calderón et al. (2018), and Pellegatti-Franco et al. (2019) used the Guenther scheme (Guenther et al., 1994; Simpson et al., 1995) to calculate online biogenic emissions. Hoshyaripour et al. (2016), Vara--Vela et al. (2018), Peralta et al. (2023), and Benavente et al. (2023) used the Model of Emissions of Gases and Aerosols from Nature (MEGAN) model (Guenther et al., 2006). The WRF-Chem v3.9.1.1 emission guide highlighted that even though the Guenther scheme is easier to run, as it does not require preparing additional input files, it has limited vegetation types which reduces the emission of important chemical species like isoprene.

In Martins et al. (2006a), biogenic emissions inventory for isoprene and terpenes were estimated by VOCs sampling based on the gradient flux method. Isoprene and terpenes were spatially distributed by RAMS forest type in the simulation domain and by types of vegetation based on the International Geosphere-Biosphere Programme (IGBP), to later simulate O_3_ using the CIT model. Alonso et al. (2010) mentioned that CCATT-BRAMS emission preprocessor used biogenic emissions from Global Emissions InitiAtive (GEIA/ACCENT) activity Databases.

In WRF-Chem dust and salt emissions are calculated online using the wind speed and land cover information. Because of the lack of measurements, an evaluation of the natural emissions calculation in the MASP have not yet been performed.

## 5 Configuration features

Fig. 2 summarizes different model configurations from our dataset. It highlights the variety of models, spatial resolutions, nested domains, and calculated performance statistics. In this section, we addressed these features in more detail.

### 5.1 Simulation periods

**Fig. 3:**
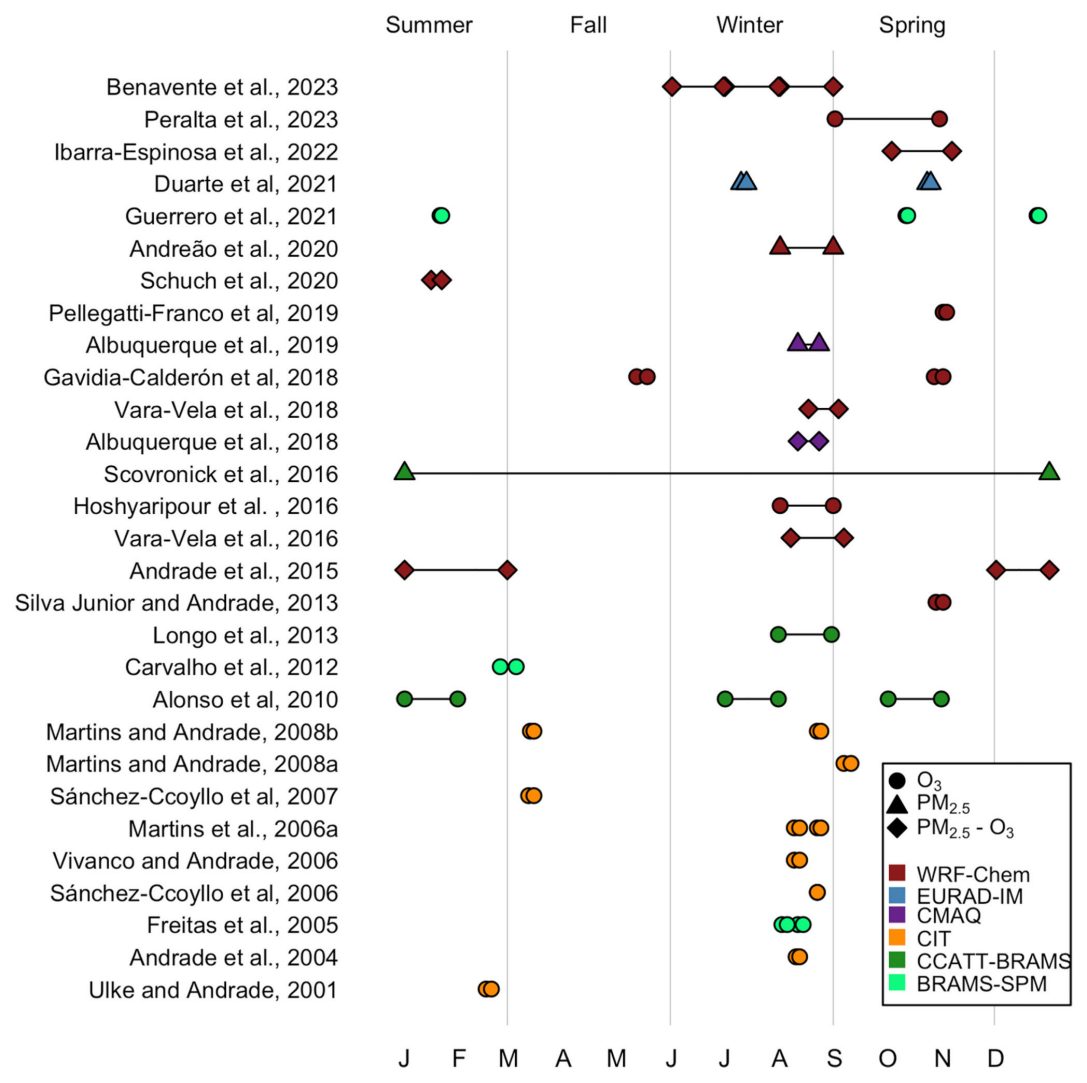
Summary of air quality modeling studies in the MASP, air quality models, simulation periods, and focused analyzed pollutants.

Fig. 3 summarizes the simulation periods for all the studies shown in Table 1. Most of the simulations (22 or 76%) are carried out at the end of winter and during spring, between the ends of August until the end of November. Biomass burning emissions also reach the MASP during September and October. This means that researchers have focused on worst case scenarios to simulate, which typically occur in spring. Days with precipitation were reported in 12 (41 %) studies.

Other simulations focused on high O_3_ concentration episodes like Carvalho et al. (2012), which can happen in summer when, despite being the wet season, solar radiation is higher and maximum hourly concentrations are recorded. Works like Gavidia-Calderón et al. (2018) and Pellegatti-Franco et al. (2019) selected their simulation periods based on the availability of ozonesondes (Andrade et al., 2012). On the other hand, to associate mortality burdens to pollution exposure, Scovronick et al. (2016) performed a full year run simulation.

Most of the simulation periods covered around three days, a typical week, or a full month. The simulation periods in early studies were very short, usually focused on pollution episodes. The increase of computer resources in the last 10 years permitted the simulation of longer periods as shown in the works of Peralta et al. (2023) and Benavente et al. (2023).

### 5.2 Domain configuration

Simulations in the MASP usually have one to three nested domains, all the simulations used at most three nested domains (Fig. 2c). The atmospheric scale most represented in these studies is the regional scale. The most used horizontal grid resolution is 5 km (13 studies), followed by 3 km (5 studies) (Fig. 2d). Only the works of Pellegatti-Franco et al. (2019) and Duarte et al. (2021) reached the local scale, as they used 1 km of grid space. Still, performing long term air quality simulation at a local scale demands a high computational cost.

Regarding the vertical resolution, simulations using the CIT model used five vertical levels. The top level’s height ranges between 1100 and 2300 m, and the first level height ranges between 20 and 80.5 m. This setup covers the planetary boundary layer (PBL), and each vertical level increases its thickness. For WRF-Chem, BRAMS-SPM, BRAMS-CCATT, and WRF (i.e. to feed the offline models), it was used over 31 levels to describe the atmosphere until the stratosphere. In those cases, strategies to describe the lower troposphere include variable spacing until 1700 m using a proportion of 1.1, and then constant separation until 19 km (Freitas et al., 2005). Alonso et al. (2010) from a first level of 100 m it increased the thickness using a geometric progression of rate 1.2. The impact of domain configuration in terms of grid space and vertical levels have not been addressed.

### 5.3 Meteorological and chemical boundary conditions

The first air quality modeling studies in the MASP used offline models. To create the meteorological fields, researchers used information from meteorological weather stations and CETESB ground stations to produce meteorological initial and boundary conditions (IC/BC). The methodology involved the spatial interpolation of these observations in the modeling domain (Andrade et al., 2004). Later, meteorological simulations from mesoscale meteorological models, like RAMS and BRAMS, produced the meteorological fields, which were run with the analysis (horizontal resolution of 1.875°) from the Center of Weather Forecast and Climate Studies of the Brazilian National Institute for Space Research (CPTEC/INPE) (Sánchez-Ccoyllo et al., 2006).

In the case of the online models from our sample, the meteorological IC/BC came from reanalysis and analysis of global meteorological models, such as the Global Forecast System (GFS) analysis (Vara-Vela et al., 2016), the European Center for Medium-Range Weather Forecasts (ECMWF) reanalysis (Hoshyaripour et al., 2016), and CPTEC/INPE analysis (Freitas et al., 2005). GFS analysis was used to create meteorological IC/BC for WRF meteorology simulation to run CMAQ (Albuquerque et al., 2018) and EURAD-IM (Duarte et al., 2021). Currently, analysis and reanalysis have a finer spatial resolution. For example, GFS analysis is available at 0.25° (https://rda.ucar.edu/datasets/ds083.3/) and has been used in Benavente et al. (2023).

For chemical initial and boundary conditions, studies with the CIT and CMAQ models used surface CETESB air quality network data. The considered pollutants were O_3_, NO_2_, SO_2_, CO, and VOCs, which were interpolated using a weighted average methodology. The Copernicus Atmospheric Monitoring Service (CAMS) was used as chemical IC/BC in the EURAD-IM model (Duarte et al., 2021). In the case of WRF-Chem, model runs used The Model of Atmospheric Transport and Chemistry-Max-Planck-Institute for Chemistry version (MATCH-MPI) runs (Silva Junior and Andrade, 2013), the default chemical IC/BC (Andrade et al., 2015), the Model for Ozone and Related Chemical Tracers, version 4 (MOZART4) model output (Gavidia-Calderón et al., 2018), and The Community Atmosphere Model with Chemistry model output (Benavente et al., 2023).

WRF-Chem has the mozbc tool to assimilate chemical IC/BC from global chemical transport models (CTM). If not used, WRF-Chem uses a default IC/BC based on a northern hemisphere clean condition simulation using the NALROM model. CCATT-BRAMS and CMAQ also count with BC-PREP and ICON BCON modules respectively to assimilate CTM results as chemical IC/BC.

Regarding the works of CMAQ in the MASP, Albuquerque et al. (2018, 2019) updated the default BC using averages from CETESB air quality stations and previous simulations test with adjusted values; the organic speciation was based on Martins et al. (2006b). For CIT simulations that also used air quality station and measurement data, the surface BC was repeated for the five vertical levels and for its lateral boundaries. In these studies, the species considered were NO_2_, NO, O_3_, VOC, and SO_2_. Andrade et al. (2004) also considered BC for aldehydes, formaldehyde, and methyl ethyl ketone.

The main challenge to create the chemical IC/BC is to map the chemical species from CTM or observations to those used in the chemical mechanism and aerosol module in the regional air quality. If the selected chemical mechanism is different from the global CTM a remapping is required, and this remapping may be a source of errors. Still, an evaluation of different global CTM simulations is mandatory to select better chemical IC/BC for the MASP and other South American cities.

Finally, to reduce the impact of initial conditions a spin-up time is usually discarded from the total simulation period. Our sample showed that for simulation periods of around one month, more than 10 days were considered for spin-up (Longo et al., 2013), meanwhile, for simulations of a week to three days, one day of spin-up was considered (Sánchez-Ccoyllo et al., 2007). Other studies used 3 days (Gavidia-Calderón et al., 2018) and 2 days (Ibarra-Espinosa et al., 2022; Vara-Vela et al., 2018) as spin-up time (Fig. 3h). Peralta et al. (2023) is the first work to run with updated meteorology IC/BC each five days of simulation. There is no consensus in the effect of spin-up days for air quality simulation for both gases and particulate matter.

### 5.4 Chemical mechanism, aerosol modules, and photolysis schemes

The chemical mechanism is the component of the air quality model that describes the pollutant chemistry. It includes the pollutant reaction pathways and kinetics (Kaduwela et al., 2015). Table 2 shows the chemical mechanisms that have been used in the air quality modeling studies described here (Fig. 2f).

Because the extensive use of ethanol in the MASP increased the emission of ethanol, all CIT simulations used the SAPRC99 chemical mechanism extended to explicitly describe ethanol, methane, methanol, isoprene, H_2_O_2_, and SO_2_. Likewise, the CBMZ mechanism was chosen to perform the air quality forecast in MASP with WRF-Chem for its inclusion of ethanol explicitly (Andrade et al., 2015).

Researchers also use different aerosol modules to simulate fine and coarse particulate matter (Table 3). They used a sectional aerosol scheme as the Model for Simulating Aerosol Interactions and Chemistry (MOSAIC, Zaveri et al., 2008); a bulk aerosol scheme like Goddard Chemistry Aerosol Radiation and Transport (GOCART, Chin et al., 2000); and a modal aerosol scheme like the Modal Aerosol Dynamics model Europe/Volatility Basis Set (MADE-VBS, Ahmadov et al., 2012). Because organic mass represents around 40 % of PM_2.5_ (Brito et al., 2013), more complex aerosol modules that account for primary (POA) and secondary organic aerosol (SOA) have been tested. For example, Vara-Vela et al. (2018) used MADE-VBS to include SOA and processes like aerosol aging.

Photolysis schemes are required to calculate the photolysis rate coefficients (Real and Sartelet, 2011). In earlier air quality models these coefficients were calculated based on pre-calculated look-up tables for assumed clear-sky condition. In this approach, spatial and temporal attenuation factors are used to account for aerosol and clouds. This was the approach used in the simulation using CIT in the MASP. For instance, Andrade et al. (2004) used a correction to account for cloud coverage based on Holtslag and van Ulden (1983). In other works, clear sky conditions were assumed, and photolysis rates were calculated using Peterson (1976) actinic fluxes estimations. In the works with CMAQ, Albuquerque et al. (2018) use JPROC that produces look-up tables for clear sky conditions, it is recalculated each simulation day and includes cloud cover correction.

Online models like CCATT-BRAMS and WRF-Chem have online calculations of photolysis rates that account for clouds and aerosols. Simulations using CCATT-BRAMS used the Fast-TUV based on Madronich (1987). In the simulations with WRF-Chem, researchers have used Madronich (1987), Fast-J (Wild et al., 2000), and Fast-TUV. In these models, the photolysis scheme is typically linked to the aerosol modules. The concentration of cloud droplets is predicted based on activated aerosols within the microphysics schemes. This information then serves as input for the shortwave radiation schemes, thereby affecting the cloud’s optical depth. Consequently, in the presence of clouds, photolysis rates of gas species below the cloud base can be attenuated (e.g. Fast et al., 2006). Even though their importance is in ozone formation and in secondary aerosol formation, there has not been any evaluation of the photolysis schemes.

Since the chemical mechanism and aerosol modules determine the speciation of emission inventories, the main challenge is the speciation of VOCs to the selected chemical mechanism and the speciation of PM into aerosol module species. Additional emission measurements are required to fill this gap. Furthermore, the evaluation of aerosol module that can represent SOA is of importance in the MASP. Depending on the use of the simulations, the question of the most suitable chemical mechanism and aerosol module for research or forecast is still unanswered.

### 5.5 Physics and dynamics options

In air quality models, the representation of sub-grid processes, like turbulence, affects the prediction of pollutant concentrations. Table 4 shows the parameterizations used in studies with WRF-Chem and studies that used WRF (only meteorology) simulations as input for offline models. Yonsei University parameterization (YSU) is the most used planetary boundary layer (PBL) scheme, together with Noah as land surface parameterization, and MM5 similarity for surface layer parameterization. In some cases, the selected physics options depend on each other. For example, Pellegatti-Franco et al. (2019) had to run with BouLac PBL scheme to use the Building Environment Parameterization (BEP) for urban canopy. In the case of Albuquerque et al. (2018), they chose the Pleim-Xiu surface layer and surface model to also use the Asymmetric Convective Model (ACM2 PBL) scheme.

The most used microphysics parameterization was the Purdue Lin scheme together with Morrison 2-moments. The Grell 3D ensemble was the most used cumulus scheme. The longwave radiation (LW) scheme most used was the Rapid Radiative Transfer Model (RRTM), while for shortwave radiation (SW) the most used was the Goddard scheme. In newer versions of WRF (>v3.7), as a good practice RRTMG scheme is used simultaneously for SW and LW.

WRF-Chem and CCATT-BRAMS include aerosol-radiation feedback. From our sample, Vara-Vela et al. (2016), Vara-Vela et al. (2018), and Ibarra-Espinosa et al. (2022) activated the feedback option in WRF-Chem. They found that during MASP dry-season it can reduce O_3_ concentration by 2%. Ibarra-Espinosa et al. (2022) also found that indirect effects included an increase in precipitation and PBL that produce lower pollutant concentrations.

We still require an evaluation of the impact of the physical parameterization on the air quality simulation in the MASP. Works like Misenis and Zhang (2010), where they performed an assessment of these options, are fundamental to improve the meteorology representation of the MASP and, therefore, improve the air quality simulations.

## 6 Model performance

Many sources of errors exist in the air quality models. Zhang et al. (2012a) and Baklanov and Zhang (2020) summarized the following errors: representation of planetary boundary layer height and atmospheric turbulence, chemical boundary conditions, uncertainties in emissions, and limited knowledge of the treatment of chemical processes of urban chemistry such in SOA formation. Therefore, thorough model evaluation must be conducted to determine if model results are fit for their intended purpose (McNider and Pour-Biazar, 2020; Rao et al., 2020).

The comparison of model results against observation is also a source of irreducible uncertainty as it involves the comparison of volume averages against point measured data (Rao et al., 2020). It is important to consider that more than one air quality station (AQS) can be located in the same grid cell, which is more probable when using lower spatial resolution. Furthermore, the model performance is calculated based on a limited number of grid points that depends on the number of AQS. Because a denser number of air quality stations are installed in the most urbanized part of the city, the model performance is mainly representative for that urban area (Swall and Foley, 2009).

There are four types of model evaluation: operational, diagnostic, dynamic, and probabilistic (Dennis et al., 2010; Seigneur et al., 2000; Simon et al., 2012). Operational evaluation compares model output against routine observations, while the diagnostic focuses on evaluating the effect of a specific process in the model results; the dynamic evaluation detects the model response to perturbations such as in meteorology conditions and emission scenarios. Probabilistic evaluation aims to estimate the level of confidence (uncertainty) in the model results. In our dataset, the main type of model evaluation included operational evaluation, diagnostic evaluation, and dynamic evaluation. Probabilistic evaluation was not performed.

From our sample of 29 studies, only 20 (69%) registered model performance statistics. To increase the sample, if a study performed two simulation periods, we count each simulation period as one observation (Simon et al., 2012). We aggregated the statistics of air quality station’s individual performance statistics for each simulation period. The performance statistics considered were calculated based on hourly concentration simulations from the inner domain. None of the selected studies that deal with air quality forecasts performed bias correction methodologies (e.g. Kalman filters). All works point out that errors in emission inventory and in the representation of meteorology are the main source of model errors.

Different studies used different performance statistics. The formulas to calculate these statistics are available in Table S1 in Supplementary Material. Pearson correlation (R), mean bias (MB), and root mean square error (RMSE) were the most common model evaluation performance statistics (Fig. 2e). These performance statistics were also found as the most used in Simon et al. (2012).

### 6.1 Ozone

To compare model performance, we first transform the units to ppb using a conversion factor of 1 ppb = 1.96 μg m^−3^ (25 °C and 1013 mb). When compared against recommendations from Emery et al. (2017), we found that all the studies reach the criteria benchmark for the Pearson correlation coefficient (R > 0.5) and more than half of the studies reach the goal criteria (R > 0.75). In the case of the normalized mean bias (NMB), seven simulations are in the criteria benchmark zone (<± 15 %); meanwhile, only two simulations reached the normalized mean error (NME) criteria benchmark (<25 %). In O_3_ modeling studies, the results are hourly concentrations (not the MDA8 concentrations). Martins and Andrade (2008b) used cut-off values of 60 ppb and 40 ppb for spring and summer simulations respectively, and Peralta et al. (2023) used cut-off values of 40 ppb.

**Fig. 4:**
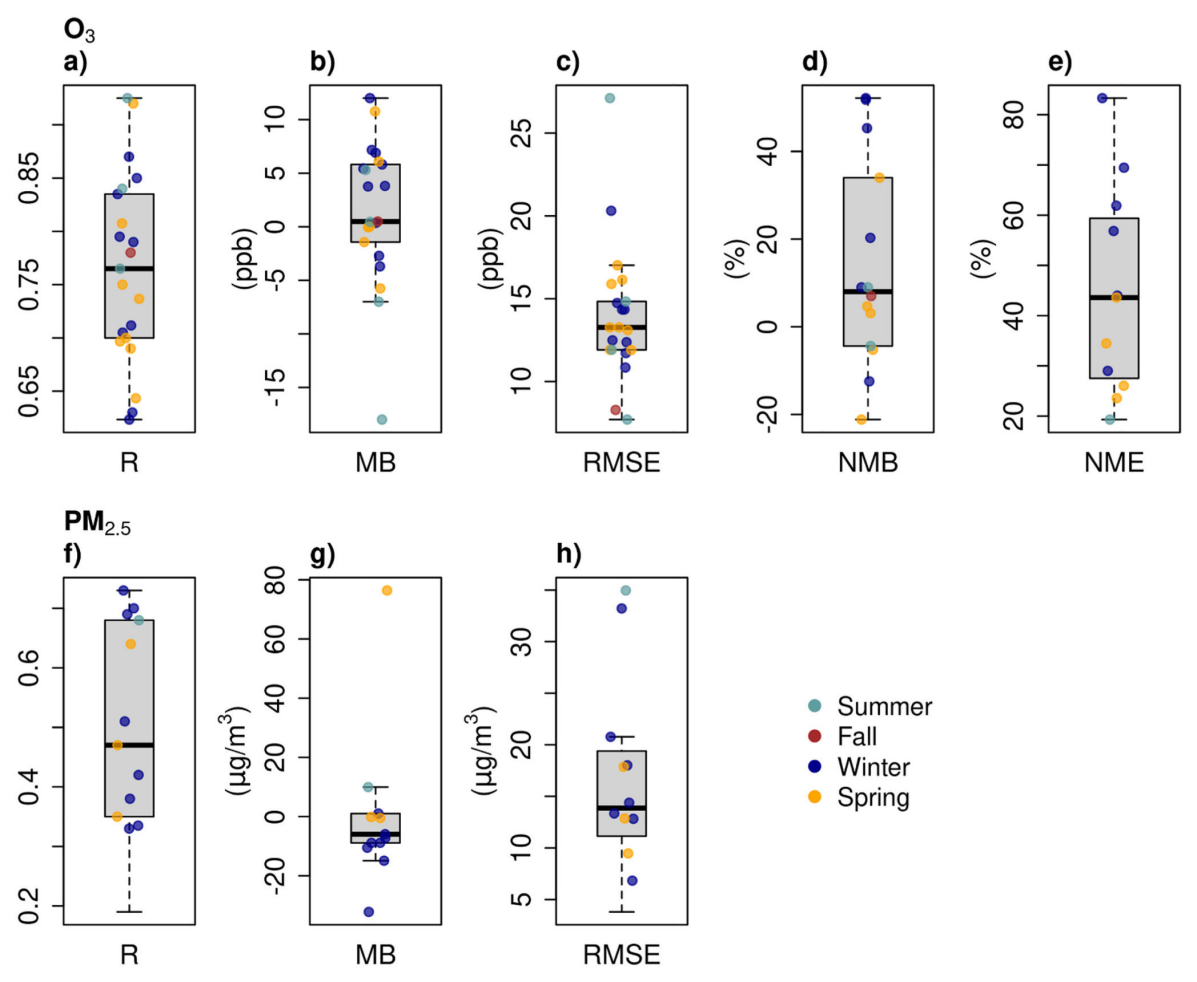
Distribution of air quality model performance statistics. Pearson correlation (R), Mean bias (MB), Root mean square error (RMSE), Normalized mean bias (NMB), and Normalized Mean Error (NME).

The Mean bias (MB) median is around zero which means that half of the studies overestimated O_3_ while the other half underestimated O_3_ concentration. The R values ranged from 0.62 to 0.93, the MB values from –18 ppb to 12 ppb, and the RMSE from 7.7 to 27.1 ppb (Fig. 4a to e). The model performance does not depend on the simulated season.

One of the causes of the overestimation of ozone concentrations is the overestimation of nocturnal ozone concentration. Gavidia-Calderón et al. (2018) and Vara-Vela et al. (2018) found that the underestimation of nocturnal NO_X_ emissions reduced O_3_ titration during the night, avoiding the consumption of O_3_. CIT simulations (Andrade et al., 2004; Vivanco and Andrade, 2006) showed that ozone underprediction was mainly caused by the overestimation of NO_X_ emission. The spatial and temporal distribution also affects the performance, as it is based on proxies and assumes the same temporal distribution of emission in every grid cell (Andrade et al., 2015). As noted by Harrison (2018), problems in temporal and spatial distribution can create bigger errors than problems in underestimation or overestimation of emissions, especially in finer spatial resolution domains. Additionally, the speciation of VOCs in the chemical mechanism is also a source of error that has not been extensively evaluated.

### 6.2 PM_2.5_

From eleven studies that evaluated PM_2.5_, only nine reported performance statistics. Only two simulations reached the R goal benchmark on PM_2.5_ (R > 0.7). NMB was used in only four studies and their values ranged from 4.30 % to 50.60 %, with only two studies reaching the Emery et al. (2017) NMB criteria benchmark (NMB within ±30 %). NME (three studies) values ranged from 40.44% to 68.94%, and only one work reached the NME criteria benchmark (NME <50 %). R values ranged from 0.19 to 0.73, MB values from −32.2 to 76.4 μg m^−3^, and RMSE values from 3.8 to 35 μg m^−3^ (Fig. 4f to h). Like in the case of O_3_ simulations, the model performance is independent of the simulated season.

In the case of the representation of PM_2.5_, the underestimation of concentrations is mainly caused by not considering all emitted sources, as well as uncertainties in the current treatments of secondary organic aerosol (SOA) formation in models. Regarding the missing sources – primarily from industrial and residential sectors - they are not spatially and temporally distributed, while the total amounts provided by CETESB are mostly outdated. Furthermore, including the biomass burning emission from FINN or 3BBM also add another layer of uncertainty (e.g. Vara-Vela et al., 2018). On the other hand, there are classes of SOA precursors that have yet to be included in models. However, even if SOA formation processes were accurately described in terms of the full set of underlying reactions, it would likely be infeasible within models due to the high computational costs involved.

## 7 Purposes and types of air quality applications

### 7.1 Model evaluation

Andrade et al. (2004) evaluated the CIT model performance to check the vehicular emissions reduction on O_3_ concentrations. This work implemented CIT to perform air quality simulations over the MASP. They found that when using the official emission inventory, O_3_ concentrations were lower than observations. They used NO_X_/CO concentration ratio to estimate the correct emission ratio from the total emissions of the official inventory. Reducing NO_X_ emissions by half yielded better results, suggesting an overestimation of NO_X_ emission in the official inventory.

Silvia Junior and Andrade (2013) evaluated WRF-Chem performance to simulate O_3_ and CO concentrations. This study is the first implementation of WRF-Chem to simulate air quality over the MASP. After the emissions were spatially and temporally calibrated, the model produced O_3_ and CO in good agreement with observations.

Longo et al. (2013) evaluated the performance of CCATT-BRAMS at different scales. They simulated air quality in the MASP at a local scale. The model showed an underestimation of CO and an overestimation of NO_X_. NO_X_ and O_3_ simulations were closer to measurements at the countryside air quality stations.

Albuquerque et al. (2018) evaluated the performance of WRF-SMOKE-CMAQ modeling system in representing meteorology and air quality. The simulations showed underprediction of PM_2.5_. The model produced NH_4_, black carbon, and NO_3_ concentration close to observations. The authors reported WRF limitation to represent rainfall and the overestimation of wind speed. Air quality performance reported below the expected desired value. The performance of O_3_ and atmospheric aerosols were acceptable.

Andrade et al. (2015) implemented the air quality forecast system (AQF) over Southeast Brazil. The authors detailed the WRF-Chem and BRAMS-SPM methodologies to implement the AQF. They showed a new spatial distribution of vehicular emissions based on road lengths. The authors highlighted that the AQF is useful to authorities and the community concerned with the impact of regulatory pollutants on health.

Duarte et al. (2021) evaluated the EURAD-IM model to simulate aerosol concentration and local and long-range transport sources. EURAD-IM produced a good PM_10_ simulation with a correlation above 0.7.

Hoshyaripour et al. (2016) compared WRF-Chem, the deterministic model, against a statistical model. Results showed that WRF-Chem better simulated O_3_ daily mean and peak concentrations. The advantage of the statistical model is its runtime velocity and a good representation of O_3_ daily mean. The author also used data from the Measurement of Pollution in the Troposphere (MOPITT) satellite instrument to spatially evaluate WRF-Chem simulation. They compared the CO column (mol cm^−3^) from MOPITT against WRF-Chem estimates for coarse and inner domains.

Finally, Benavente et al. (2023) evaluate WRF-Chem simulation using satellite information, together with observation from a mobile station, and CETESB air quality stations. This work showed a methodology to quantitatively evaluate the simulated pollutant concentrations with satellite data retrieved from the MOPITT, the Moderate resolution Imaging Spectroradiometer (MODIS), and the Ozone Monitoring Instrument (OMI) sensors.

### 7.2 Model development

Ulke and Andrade et al. (2004) improved the method to calculate turbulent diffusion on the CIT model. The new implementation produced a better representation of turbulence inside the PBL. It produces higher O_3_ concentrations closer to observations.

Freitas et al. (2005) developed a simplified and operational photochemical model, the BRAMS-SPM. It consisted of a simplified photochemical module in the RAMS mesoscale model. Results showed a good correlation between observations even in representing nocturnal O_3_ concentrations. BRAMS-SPM is suitable for operational air quality forecasts.

### 7.3 Impact of model inputs

Gavidia-Calderón et al. (2018) evaluated the impact of using dynamical boundary conditions on the representation of O_3_ concentration with WRF-Chem. They used MOZART4 as chemical background concentrations. They found that the impact on O_3_ simulation was higher during periods of lower photochemical activity (during Fall), and the impact was lower during spring. It improved the representation of nocturnal O_3_ and the O_3_ vertical profile.

Pellegatti-Franco et al. (2019) improved the land-cover information by assimilating World Urban Database and Access Portal Tools (WUDAPT) information into WRF-Chem. They simulated O_3_ using three nested domains with the inner domain of 1 km of spatial resolution. Even when there was an improvement in the meteorological representation, especially in wind speed, the O_3_ concentration was worse when using the improved land-use configuration. This suggests that there is an error compensation in air quality models where errors in emission inventory sometimes are corrected by errors in the meteorology part.

### 7.4 Evaluation of emission inventories

Alonso et al. (2010) developed a vehicular emission for South America, they distributed emissions estimates from EDGAR and REanalysis of the TROpospheric chemical composition (RETRO) emission inventories based on an algorithm that delimits urban areas using remote sensing data. It avoids representing cities that are close to each other as a single urban area. They highlighted the need to include monthly and diurnal variability (day of the week) to improve the O_3_ estimated when running CCATT-BRAMS simulations.

Recently, Andreão et al. (2020) evaluated emission estimates of PM based on the Brazilian top-down vehicle emission inventory. PM emissions were spatially distributed based on the population and the vehicular fleet of each evaluated city. EDGAR emission inventory was used for comparison. WRF-Chem results showed that using EDGAR produced higher PM concentrations because EDGAR includes other emissions sources than vehicle emissions. The proposed spatially distributed inventory produced better results.

### 7.5 Impact of emission scenarios

Vivanco and Andrade (2006) evaluated the official NO_X_ and VOC vehicular emission estimates from CETESB. They used the concentration of CO and NO_X_ during 7 a.m. and 8 a.m. (local time) to correct vehicular emission estimates. It assumed that the lower reactivity of these pollutants occurs in the morning hours, and that CO was correctly measured. They found that NO_X_ emission was 0.5 times lower and VOCs emissions 1.2 higher than CETESB estimates.

Sánchez-Ccoyllo et al. (2007) evaluated the O_3_ sensitivity to precursors from different emission scenarios. They found that using pre-1989 technology vehicular emissions produced the worst air quality scenario. When using policies to control emissions (i.e PROCONVE), lower O_3_ concentrations are achieved. In this case, the CIT model reported problems in simulating nocturnal O_3_ concentrations.

Scovronick et al. (2016) used CCATT-BRAMS to estimate the effects on air quality and health of ethanol fuel scenario and of gasoline fuel scenario. The gasoline scenario led to a reduction of PM_2.5_ and O_3_ concentrations which reflected a reduction in mortality. The authors recommended that new emissions regulations on ethanol must be addressed.

Albuquerque et al. (2019) evaluated emission control strategies to reduce PM_2.5_ concentrations using the WRF-SMOKE-CMAQ modeling system. The authors evaluated a baseline scenario and different emissions scenarios reducing gas emissions of SO_2_ and NH_3_, and particle emissions of sulfate (SO_4_) and nitrate (NO_3_). Reducing SO_2_ is not an effective strategy. Reduction in PM_2.5_ concentration is not related to the same emission reduction ratio. Reducing 50% of NH_3_, SO_2_ and NO_X_ lead to a bigger reduction. SOA and black carbon need to be addressed in policy strategies as they formed 70% of PM_2.5_.

Schuch et al. (2020) estimated changes in O_3_ and PM_2.5_ under emissions scenarios from ECLIPSEv5a: Current legislation (CLE), mitigation, maximum feasible reduction (MFR) under Representative Concentration Pathway (RCP4.5) climate scenario using WRF-Chem. MFR produced cleaner air with a reduction of 3%–75% of O_3_ and PM_2.5_ respectively. CLE increased O_3_ and PM_2.5_ concentrations by 1% and 11% respectively.

### 7.6 Ozone formation

Martins et al. (2006a) estimated the impact of using Biogenic VOCs (BVOCs) on O_3_ formation using the CIT model. Emissions that included BVOC emissions (isoprene and terpenes) produced 15% more O_3_. Martins and Andrade (2008a) evaluated the impact of different VOC species on O_3_ formation. Simulations are more sensitive to VOCs emission than NO_X_, determining that the MASP presents a VOC-limited regime. Principal species that affect O_3_ formation were aromatics, olefins, ethene, and formaldehyde.

Martins and Andrade (2008b) evaluated the impact of the reformulation of gasohol and ethanol on O_3_ formation. This is the first work that used speciation from vehicle exhaust from tunnel measurements. The scenario where all light vehicles run on pure ethanol improved air quality.

Guerrero et al. (2021) used BRAMS-SPM to study the formation of nocturnal O_3_ peaks in the MASP. They found that nocturnal O_3_ peaks are more related to vertical transport from higher levels to the ground than synoptic conditions.

### 7.7 PM_2.5_ formation

Vara-Vela et al. (2016) estimated the impact of vehicular emission on PM_2.5_ concentrations using WRF-Chem. The reaction of primary gases resulted in the formation of secondary particles that represented 20–30% of PM_2.5_ mass. Hydrocarbons produced 4% of PM_2.5_ mass. Feedback activation produced a reduction of 2% in O_3_ concentration. Later, Vara-Vela et al. (2018) estimated the impact of biomass burning emissions on aerosol concentration and properties. During long-range transport of biomass burning products, PM_2.5_ and O_3_ concentrations are 15 μg m^−3^ (24%) and 26 μg m^−3^ (32%) higher. Biomass burning is responsible for 20% of baseline particle number concentration. In both works, the authors showed the versatility of models when changing the default model aerosol bins to match the aerosol bins of the analyzer.

### 7.8 Meteorology and air pollution interactions

Sánchez-Ccoyllo et al. (2006), using the CIT model estimated the impact of meteorological variables and emissions regimes on O_3_ formation. They found that wind speed, PBL height, and air temperature influenced the most in O_3_ concentration peaks. As the reduction of VOC emissions lead to a reduction in O_3_ formation, the authors concluded that the MASP presented a VOC-limited regime.

Carvalho et al. (2012) studied a high O_3_ episode using BRAMS-SPM. They found that weak wind during the night and during the early morning accumulates O_3_ precursors, the timing of sea breeze also impacts O_3_ formation and was correctly simulated by the model.

Ibarra-Espinosa et al. (2022) evaluated the impact of vehicular emissions on meteorology and air quality. Aerosol feedback was activated when running WRF-Chem. The aerosol feedback is stronger during the wet period. During the dry season, the aerosol effect reduced 1.3% solar radiation and 1.5% O_3_ concentration. The indirect effect increases precipitation, increases PBL height, and therefore reduces pollutant concentration.

Peralta et al. (2023) studied the impact of atmospheric conditions from RCP scenario 4.5 and 8.5 on O_3_ formation for the year 2030. Results showed higher peak O_3_ concentrations on both scenarios, being the RCP 8.5 scenario the one with the higher values (5.92 % more). Nevertheless, precipitation registered in days in scenario RCP 8.5 could lead to lower O_3_ concentration.

## 8 Challenges and limitations

Researchers pointed out that uncertainties in the emission inventory and errors in the representation of meteorology are the main causes of low model performance. This is mainly caused by the limited information available on pollutant measurements, emission inventories, and urban meteorology measurements in the MASP.

When building the emission file, limited information on different sources other than vehicular emissions avoids accounting for the complete sources of air pollutants in the MASP. In the case of industrial emissions, as noted before, the information is outdated and reported in totals. For biogenic and biomass burning emissions, although they can be estimated through modeling, still there are not enough emissions measurements to evaluate the accuracy of their estimates.

The lack of information also limited the model evaluation. The pollutant observations for comparison came from the CETESB air quality network (See Supplementary Material). Unfortunately, measurements of VOCs concentration are not available, and the analysis of the model’s performance regarding other precursors of O_3_ and SOA is also limited. The same happened with the simulation of meteorological conditions, which are mainly evaluated in terms of temperature, relative humidity, wind speed and direction. Other meteorological parameters that affect pollutant concentrations, such as radiation and PBL height, have not been fully evaluated. Additionally, since all air quality stations are located in urban areas, we have insufficient knowledge about the performance of models in rural areas. Works like Squizzato et al. (2021) and Benavente et al. (2023) can help to reduce this gap as they used a mobile air quality monitoring station to cover rural areas without air quality and satellite data respectively.

Despite the lack of information, researchers have made use of measurement campaigns performed in the 23 years interval of this work. When available, researchers have used information from ozone soundings, lidar, and aircraft measurements (Freitas et al., 2005; Gavidia-Calderón et al., 2018; Pellegatti-Franco et al., 2019; Vara-Vela et al., 2018). Moreover, there have been four tunnel experiments in the MASP that provided new emissions factors, vehicle counts, VOCs and aerosol composition and speciation (Sánchez-Ccoyllo et al., 2009; Pérez-Martinez et al., 2014; Nogueira et al., 2021). This information helped to improve official emission factors, estimate emission temporal profiles, and set up VOC and aerosol speciation for chemical and aerosol mechanisms. Also, to better represent the vehicular emissions spatial distribution, different proxies have been tested, from land use categories to nighttime lights and street length. These methods are reflected in the development of emission tools for the MASP, such as AAS4WRF (Vara-Vela et al., 2017) and VEIN (Ibarra-Espinosa et al., 2018), which have been used in other cities than the MASP (González et al., 2018; Pinto et al., 2020).

To improve the meteorological representation, researchers have updated meteorological input data in the air quality models. For instance, instead of only relying on surface meteorological stations for initializing the IC/BC, they have transitioned to using global meteorological reanalysis with a higher spatial resolution, reaching up to 0.25° (Benavente et al., 2023). Efforts to update the urban parameterization in models have also been made (Pellegatti-Franco et al., 2019), but even when having a better urban classification, lack of geomorphological and radiative parameters (e.g building height, roof width, road with, anthropogenic heat, etc) for each urban class still need to be estimated and refined.

Another limitation was the computer resources. One of the reasons for short-period simulation times was the limited computational resources. Simulating entire months or even years of O_3_ and PM_2.5_ posed significant challenges, particularly when incorporating feedback mechanisms. In the case of online air quality models, such as WRF-Chem, running air quality simulations required approximately five times the computational time needed for meteorological simulations alone. If the model involved interactions with weather patterns and advanced aerosol chemistry, the computational cost could escalate up to 10 to 100 times a standalone WRF simulation (Ahmadov et al., 2018). Fortunately, the lower cost of computational resources, and collaboration with other institutions’ supercomputers, will allow more detailed air quality simulations: higher spatial resolution, more complex chemical mechanisms, aerosol modules, and feedback between the chemistry and meteorology.

## 9 Perspectives and recommendations

We identified the following areas of research that have not been tackled yet:

- Given that the MASP is one of the most populated cities in the region, assessing the impact of climate change on air quality is mandatory. Therefore, it is crucial to investigate the implications of various climate scenarios, including the Representative Concentration Pathways (RCP) and the emerging Shared Socioeconomic Pathways (SSP), on the concentration of O_3_ and PM_2.5_. Especially when these pollutants have not decreased their concentration despite of the emission mitigation policies.
- It is important to use air quality models to study the impact of the pollutants on health in the MASP. Air quality models provide high-resolution pollutant concentration that facilitates the analysis of spatio-temporal variability of pollutant concentration. This allows a better association between health effects and air quality and exposure estimates (Rao et al., 2011). In addition, air quality models through the manipulation of the emission file allow linking the health effects to a specific emission source and even to a specific pollutant (Gao et al., 2018). Previous works that studied the effect of air pollution on health (Costa et al., 2017; Santana et al., 2020), only relied on CETESB data.
- Satellite data can also improve model input data (Vijayaraghavan et al., 2008). In the MASP, they can be used to update land use data and vegetation types, which are usually outdated datasets in the models. Inversion modeling with satellite data to improve emission inventories, e.g. Wang et al. (2020), have not been performed in the MASP and should be explored.
- As in the work of Misenis and Zhang (2010) an evaluation of the different physical parameterization must be studied in the MASP. The evaluation of different model physics parameterizations should be addressed to improve the representation of cloud, precipitation, radiation, and nocturnal boundary layer. Furthermore, urban parameterizations need to be configured and evaluated to better represent the MASP urban climate.
- Likewise, the evaluation of different chemical mechanisms, aerosol modules, and VOCs and PM speciation should also be studied to see what is suitable for research and forecasting in the MASP. Other key points in the model configuration that must be studied include the effects of photolysis schemes, the implication of activating aerosol feedback, and the performance of biogenic and biomass emission models.
- As in the MASP no modeling study used data assimilation modules (e. g. WRFDA), applying bias correction methodologies should be evaluated and implemented. These methodologies could improve the operational air quality forecast estimates.

Finally, to tackle the above challenges and to improve the air quality modeling practices in the MASP, we suggest the following recommendations:

- In the spirit of reproducible research, we recommend sharing the model configuration and the emission files in a data repository, as these inputs are fundamental to reproduce and explain the model results.
- It should be a common practice to include the model evaluation of the meteorological parameters, as they help to interpret the modeling results.
- Consequently, an emission dataset could be harnessed and perform a model intercomparison. Differences in simulated pollutant concentrations could be caused by differences in the model emission inventories. This could also guide us toward a model ensemble to forecast air quality in the MASP.
- Satellite data should be used in combination with CETESB air quality network to improve spatial model evaluation. Especially in locations with less density of air quality stations around the City of São Paulo. Satellite information can be used to check the model representation of the MASP air pollution plumes. Currently, there are more satellite databases available for different chemical species that are not measured by the CETESB that can be used in future modeling works (e.g., formaldehyde and aerosol properties from the TROPOspheric Monitoring Instrument (TROPOMI) onboard the Sentinel-5 Precursor satellite).
- We recommend using Emery et al. (2017) benchmark statistics to improve model performance intercomparison. At the same time, to compare the results with other cities’ simulations, we also recommend including the calculation of daily maximum 8-h average (MDA8) in the performance statistics. The simulated O_3_ MDA8 could also be compared with São Paulo state current legislation.
- WRF-Chem and CMAQ were implemented in the MASP to make operational air quality forecasts. Nevertheless, the forecast only consists of pollutant concentration estimations. Models should be used to provide other services like the forecast of air quality indices or alerts that are more understandable to the population based on the ones used by CETESB.

## 10 Summary and conclusions

We reviewed 29 air quality modeling studies performed over the MASP published between 2001 and 2023. These studies exemplify how air quality models, together with field experiments and observations from the air quality network, improved the understanding of the atmospheric chemistry of this megacity.

Researchers have used offline models such as CIT, CMAQ, and EURAD-IM and online models such as BRAMS-SPM, CCATT-BRAMS, and WRF-Chem. Earlier applications focused on O_3_ formation with simulation periods up to three days describing pollution episodes. In later years, simulations focused on PM_2.5_ and SOA formation, and the simulation periods were extended from a few days to complete weeks and months.

WRF-Chem was the most used model followed by the CIT model; together they represent 69 % of our dataset. The air quality modeling covered the regional scale with a most frequent spatial resolution of 5 km, which is commonly used through a three-nested domain configuration. Only two works performed a simulation of 1 km spatial resolution. Most of the simulation periods have been performed during the end of winter and the spring as higher concentrations of ozone and PM_2.5_ are recorded during these seasons.

The main source of uncertainties is the emission inventory as researchers usually recommended its improvement and calibration. Vehicular emission was mainly used as total anthropogenic emission input, and other anthropogenic sources such as industrial were completed using global emission inventories, which are not precise for South America as the information is scarce. Efforts to include industrial emissions are important. This information, therefore, should be freely available like the information from the air quality network. Biogenic emissions are usually estimated using MEGAN and biomass burning emissions using FINN. Validation of those methodologies could also improve the modeling.

Measurement campaigns must continue. Tunnel experiments are essential to improve the emission estimates by improving the emission factors and the VOCs and PM speciation required to create the emission files that depend on the chemical mechanism and aerosol module. PM composition analysis is also important to evaluate PM formation mechanisms in the model (e.g. SOA formation). On the other hand, meteorological parameters as PBL height inside the urban areas will improve the evaluation of PBL and urban physics parameterizations.

The most used performance statistics were the mean bias (MB), Pearson correlation coefficient (R), and the root mean square error (RMSE). Ozone modeling performance statistics reached Emery et al. (2017) Pearson correlation criteria benchmark (R > 0.7). PM_2.5_ simulations were not as good as O_3_ estimates. Future air quality modeling studies should follow the recommended statistics (R, NMB, and NME) from Emery et al. (2017) and include the calculation of MDA8 performance statistics to increase the intercomparison with simulation with other cities. Evaluation of new chemical mechanisms, aerosol modules, and VOC and PM speciation should be studied to see what are the most suitable for research and forecasting. Meteorological and chemical data assimilation for air quality simulations and bias correction methodologies for air quality forecast have not been applied. These techniques should be explored to improve model performance.

Many fields of application are still missing like studies on the impact of climate change on future air quality, and the impact of air pollution on population health. The use of satellite data for model evaluation as well as the use of bias correction techniques or data assimilation will improve the operational air quality forecast.

Researchers have made many efforts to implement and run air quality models in the Metropolitan Area of São Paulo. They created the emission files, tested new chemical mechanisms and aerosol modules, updated IC/BC with higher resolution data, and added information from experiment campaigns. These simulations studied the sensitivity to precursors of O_3_ and PM_2.5_, the influence of emissions scenarios, and new emissions estimations. We believe that this review provides a reference for further air quality studies over MASP. We also believe that the model configurations and strategies to distribute emission inventories can be used for other cities in the region with limited information and where the main source of air pollution is the vehicular fleet. Kumar et al. (2018) included modeling in the five steps to improve air quality, we hope that this review takes us closer to that goal.

## Supplementary Material

Appendix

## Figures and Tables

**Table 1:** MASP air quality modeling studies included in this review.

Reference	Models	Chemical mechanism/aerosol module^a^	Simulated pollutants	Seasons /Year
Ulke and Andrade (2001)	CIT	Condensed version of the LCC (Lurmann et al.,1987)	O_3_	Summer/1989
Andrade et al. (2004)	CIT	SAPRC99	O_3_, NO_x_, CO	Winter/1999
Freitas et al. (2005)	RAMS-SPM	SPM	O_3_	Winter/1999
Martins et al. (2006a)	CIT	SAPRC99	O_3_, NO_2_, NO, PAN	Winter/1999, 2000
Sánchez-Ccoyllo et al. (2006)	CIT	SAPRC99	O_3_	Winter/2000
Vivanco and Andrade (2006)	CIT	SAPRC99	O_3_, NO_x_, VOC	Winter/1999
Sánchez-Ccoyllo et al. (2007)	CIT	SAPRC99	O_3_, NO_x_, VOC, CO	Summer/2000
Martins and Andrade (2008a)	CIT	SAPRC99	O_3_	Spring/2004
Martins and Andrade (2008b)	CIT	SAPRC99	O_3_, NO_x_, VOC, CO	Fall, winter/2000
Alonso et al. (2010)	CCATT-BRAMS	RACM	O_3_, NO_x_, CO	Summer, winter, spring/2005
Carvalho et al. (2012)	BRAMS-SPM	SPM	O_3_, NO_x_	Summer/2003
Longo et al. (2013)	CCATT-BRAMS	RELACS	O_3_, NO_x_, CO	Winter/2011
Silva Junior and Andrade (2013)	WRF-Chem	RADM2/MADE-SORGAM	O_3_, CO	Spring/2006
Andrade et al. (2015)	WRF-Chem/BRAMS-SPM	CBMZ/MOSAIC-8bins	O_3_, PM_2.5_, NO_x_	Summer/2013, 2014
Vara-Vela et al. (2016)	WRF-Chem	RADM2/MADE-SORGAM	PM_2.5_, PM_10_, O_3_	Winter/2014
Hoshyaripour et al. (2016)	WRF-Chem	MOZART/GOCART	O_3_, NO_x_, VOC	Winter/2012
Scovronick et al. (2016)	CCATT-BRAMS	Not specified	PM_2.5_	Year/2019-2020
Albuquerque et al. (2018)	CMAQ	CB05/AERO4	PM_10_, PM_2.5_, O_3_, BC, SO_4_, NH_4_, NO_3_	Winter/2008
Vara-Vela et al. (2018)	WRF-Chem	CB05/MADE-VBS	O_3_, PM_2.5_, EC, BC	Winter/2014
Gavidia-Calderon et al. (2018)	WRF-Chem	CBMZ	O_3_	Fall, pring/2006
Albuquerque et al. (2019)	CMAQ	CB05/AERO4	PM_2.5_	Winter/2008
Pellegatti-Franco et al. (2019)	WRF-Chem	CBMZ	O_3_	Spring/2008
Schuch et al. (2020)	WRF-Chem	CBMZ/MADE-SORGAM	O_3_, PM_2.5_	Summer/2019
Andreao et al. (2020)	WRF-Chem	RADM2/GOCART	PM_2.5_	Winter/2015
Guerrero et al. (2021)	BRAMS-SPM	SPM	O_3_	Summer/2005, 2010, Spring/2001
Duarte et al. (2021)	EURAD-IM	RACM-MIM/MADE-SORGAM	PM_10_, PM_2.5_	Winter, spring/2006
Ibarra-Espinosa et al. (2022)	WRF-Chem	RADM2/MADE-SORGAM	PM_2.5_, O_3_	Spring/2014
Peralta et al. (2023)	WRF-Chem	CBMZ	O_3_	Spring/2018
Benavente et al. (2023)	WRF-Chem	MOZART4/GOCART	O_3_, PM_2.5_, NO_x_, CO	Winter/2017, 2018, 2019

CIT: California Institute of Technology airshed model, CMAQ: Community Multiscale Air Quality model, EURAD-IM: The European Air Pollution Dispersion and Inverse Model, WRF-Chem: The Weather Research and Forecasting model coupled with Chemistry, BRAMS-SPM: The Brazilian Development on the Regional Atmospheric Modeling System with the Simple Photochemical Module, CCATT-BRAMS: The Coupled Chemistry Aerosol-Tracer Transport model on the BRAMS.

LCC: Lurmann, Carter and Coyle mechanism; SAPRC99: California Statewide Air Pollution Research Center photochemical mechanism, SPM: Simple Photochemical Module; RACM: Regional Atmospheric Chemistry Mechanism; RACM-MIM: RACM with Mainz Isoprene Mechanism; RELACS: Regional Lumped Atmospheric Chemical Scheme; RADM2: Regional Acid Deposition Model, version 2; CBMZ: Carbon bond mechanism, version Z; MOZART4: Model for Ozone and Related Chemical Tracers, version 4; CB05: Carbon-bond mechanism, version 5.

MADE-SORGAM: Modal Aerosol Dynamics model Europe – Secondary Organic Aerosol Model; MOSAIC: Model for Simulating Aerosol Interactions and Chemistry; GOCART: Georgia Tech/Goddard Global Ozone Chemistry Aerosol Radiation and Transport model; AERO4 the fourth-generation modal CMAQ aerosol model with extensions for sea salt emissions and thermodynamics: MADE-VBS: MADE-Volatility Basis Set.

a Only CIT and BRAMS-SPM do not have an aerosol module.

**Table 2:** Chemical mechanism used in air quality model in MASP.

Chemical mechanism	Number of species	Number of reactions	Used in MASP	Reference
LCC	35	106	1	Lurmann et al. (1987)
SAPRC99	70	223	7	Carter (2000)
RACM	70	237	1	Stockwell et al. (1997)
RACM-MIM	84	244	1	Geiger et al. (2003)
SPM		15	3	Freitas et al. (2005)
RELACS	37	128	1	Crassier et al. (2000)
RADM2	63	136	4	Stockwell et al. (1990)
CBMZ	67	164	5	Zaveri and Peters (1999)
MOZART4	85	157	2	Emmons et al. (2010)
CB05	52	156	3	Sarwar et al. (2008)

LCC: Lurmann, Carter and Coyle mechanism; SAPRC99: California Statewide Air Pollution Research Center photochemical mechanism, SPM: Simple Photochemical Module; RACM: Regional Atmospheric Chemistry Mechanism; RACM-MIM: RACM with Mainz Isoprene Mechanism; RELACS: Regional Lumped Atmospheric Chemical Scheme; RADM2: Regional Acid Deposition Model, version 2; CBMZ: Carbon bond mechanism, version Z; MOZART4: Model for Ozone and Related Chemical Tracers, version 4; CB05: Carbon-bond mechanism, version 5.

**Table 3:** Aerosol modules used in air quality simulation in MASP.

Aerosol module	Scheme	Solve SOA	Used in MASP	Reference
MADE-SORGAM	Modal	YES	4	Schell et al. (2001)
MOSAIC-8bins	Sectional	NO	1	Zaveri et al. (2008)
GOCART	Bulk	NO	3	Chin et al. (2000)
AERO4	Modal	YES	2	Binkowski and Roselle (2003)
MADE-VBS	Modal	YES	1	Ahmadov et al. (2012)

MADE-SORGAM: Modal Aerosol Dynamics model Europe - Secondary Organic Aerosol Model; MOSAIC: Model for Simulating Aerosol Interactions and Chemistry; GOCART: Georgia Tech/Goddard Global Ozone Chemistry Aerosol Radiation and Transport model; AERO4 the fourth-generation modal CMAQ aerosol model with extensions for sea salt emissions and thermodynamics: MADE-VBS: MADE-Volatility Basis Set.

**Table 4:** PBL, Land surface, and surface layer parameterizations in WRF and WRF-Chem simulation.

Reference	PBL	Land surface	Surface Layer	Microphysics	Longwave radiation	Shortwave radiation	Cumulus
Silva Junior and Andrade (2013)	MYJ	Noah	Eta similarity	Purdue Lin	RRTM	Dudhia	Grell 3D ensemble
Andrade et al. (2015)	YSU	Noah	–	WRF single-moment 5-class scheme	RRTM	Goddard	Grell 3D ensemble
Hoshyaripour et al. (2016)	YSU	Noah	–	Morrison 2-moments	RRTM	Goddard	Grell 3D ensemble
Vara-Vela et al. (2016)	YSU	Noah	MM5 similarity	Purdue Lin	RRTM	Goddard	Grelll 3D ensemble
Gavidia-Calderon et al. (2018)	YSU	Noah	MM5 similarity	Purdue Lin	RRTM	Goddard	Grell 3D ensemble
Albuquerque et al. (2018) ^a^	ACM2 (Pleim)	Surface model Pleim-Xu	Pleim-Xu	Thompson	RRTM	Dudhia	Kain-Fritsch (new ETA)
Vara-Vela et al. (2018)	YSU	Noah	MM5 similarity	Morrison 2-moments	RRTMG	RRTMG	Multiscale Kain- Fritsch
Albuquerque et al. (2019) ^a^	ACM2 (Pleim)	Surface model Pleim-Xu	Pleim-Xu	Thompson	RRTM	Dudhia	Kain-Fritsch (new ETA)
Pellegatti-Franco et al. (2019)	Boulac	Noah	Eta similarity	Purdue Lin	RRTMG	RRTMG	
Andreao et al. (2020)	YSU	Noah	MM5 similarity	Morrison 2-moments	RRTMG	RRTMG	Multiscale Kain- Fritsch
Schuch et al. (2020)	YSU	Noah	MM5 similarity	Morrison 2-moments	RRTMG	RRTMG	Grell 3D ensemble
Duarte et al. (2021) ^a^	YSU	Noah	–	WRF Single-Moment 3-class	RRTM	Dudhia	Grell 3D ensemble
Ibarra-Espinosa et al. (2022)	YSU	Noah	MM5 similarity	Purdue Lin	RRTM	New Goddard	Grell 3D ensemble
Peralta et al. (2023)	BouLac	Noah	MM5 similarity	Morrison 2-moments	RRTM	RRTMG	Grell 3D ensemble
Benavente et al. (2023)	YSU	Noah	MM5 similarity	Morrison 2-moments	RRTMG	RRTMG	Grell 3D ensemble

MYJ: Mellor-Yamada-Janjic; YSU: Yonsei University; ACM2: Asymmetric Convective Model; BouLac: Bougeault-Lacarrère; RRTM: Rapid Radiative Transfer Model, RRTMG: RRTM for general circulation models (GCM).

a Offline models that used WRF simulation as meteorological input.

## Data Availability

Data will be made available on request.

## Highlights

- Review of air quality modeling in the Metropolitan area of São Paulo from 2001 to 2022.
- Strategies for urban air quality simulation impacted by vehicular emissions.
- Analysis of air quality model performance for O_3_ and PM_2.5_ simulations.
