# Supplementary material for: Air quality modeling in the metropolitan area of São Paulo, Brazil: A review
Source: Atmos Environ (1994). Author manuscript; Available in PMC 2024 Jun 1. (PMC7616053; doi:10.1016/j.atmosenv.2023.120301)
Supplement: Appendix [file EMS196104-supplement-Appendix.docx]

**Supplementary material**

### Model descriptions

### Offline models

#### CIT

The California Institute of Technology (CIT) airshed model (McRae et al., 1982; McRae, G. J., & Seinfeld, 1983) was the first Eulerian model applied and evaluated in the MASP to simulate O_3_ concentrations. It was designed to calculate reactivate gases concentrations inside the planetary boundary layer (PBL). It is formed by the meteorology, chemistry, and emission inventory modules. CIT used the 1999 California Statewide Air Pollution Research Center (SAPRC99) chemical mechanism (Carter, 2000) to explicitly calculate organic and inorganic species and lump organic species. CIT does not have an aerosol module.

####

#### CMAQ

The Community Multiscale Air Quality (CMAQ) model is a modeling system developed by United State Environmental Protection Agency (USEPA). It was designed to treat interactions between different atmospheric scales and to evaluate the impacts of pollutant concentrations. It can simulate both reactive gases and aerosol concentrations. It was developed to be applied from regulatory and policy analysis to understanding atmospheric chemistry and physics across spatial scales ranging from local to hemispheric (Byun & Shere, 2006).

#### EURAD-IM

The European Air Pollution Dispersion and Inverse Model (EURAD-IM) system is an air quality forecast model in its forward simulation and data assimilation and inverse modeling tool in its backward adjoint run. It has nested capabilities that reach 1 km of spatial resolution. It simulates reactive gases and aerosol concentrations. It also has an emission module to process emission inventories. It presents multiple chemical mechanisms and parameterizations (Elbern et al., 2007).

### Online models

#### WRF-Chem

The Weather Research and Forecasting model with Chemistry (Grell et al., 2005) is a widely used multiscale air quality model. The meteorological module is the WRF-ARW model (Skamarock et al., 2019). Its chemical module can simulate both gas chemical reactions and aerosol formation, including feedback between chemical species and meteorological parameters (e.g. radiative feedback). It has many physical parameterizations and chemical mechanisms. The users can include different emission sources: anthropogenic, biogenic, fire emissions, sea salt, and dust emissions.

####

#### BRAMS-SPM

The Brazilian Development on the Regional Atmospheric Modeling System (BRAMS) with the Simple Photochemical Module (SPM), originally named RAMS-SPM, was designed to be an operational forecast model for O_3_ concentration (Freitas et al. 2005). The meteorological module is based on BRAMS, which is based on RAMS atmospheric model (Pielke et al., 1992). The SPM mechanism is a simplification of the SAPRC99 chemical mechanism. SPM considers 10 inorganic and 5 organic reactions without considering VOCs speciation to calculate ozone concentrations. As its focus was operationality, it is computationally efficient and can be run on small clusters. It also includes the Town Energy Budget model to represent urban areas. BRAMS-SPM does not have an aerosol module.

####

#### CCATT-BRAMS

The Coupled Chemistry Aerosol-Tracer Transport model on the BRAMS (CCATT-BRAMS) is a regional air quality model developed to integrate weather and air quality forecasting and research. CCATT-BRAMS represents gaseous and aqueous chemistry and aerosol formation. Its formulation includes chemical formation, transport, removal, and dispersion of pollutants (Freitas et al, 2009; Longo et al., 2013).

### CETESB Air quality network

The CETESB air quality network is one of the most extensive networks in South America with around 61 air automatic stations located in the State of São Paulo (Riojas-Rodríguez et al., 2016). It also has 22 manual stations and two mobile stations (CETESB, 2021). This air quality network provides information to perform air quality model evaluation of regulatory pollutants such as O_3_, NO, NO_2_, CO, PM_2.5_, and PM_10_. The air quality stations also measure meteorological parameters such as temperature, relative humidity, wind speed and direction. All of the modeling studies of the MASP have used this data to evaluate their model results. The information is freely available through the QUALAR system, which also provides real-time data (<https://cetesb.sp.gov.br/ar/qualar/>). Ozone and regulatory pollutants such as CO, SO_2_, and PM have been measured since 1982 and PM_2.5_ since 2011 using automatic analyzers (Andrade et al. 2017).

Some of the limitations of the network are that the air quality stations are clustered inside the City of São Paulo, and they are installed in urbanized areas, even the ones located outside the MASP (Fig 1b). Another limitation is that not all the stations measure all the regulated pollutants, and not all the stations have complete meteorological data. Finally, the network does not have a well-defined background monitoring station. Pico do Jaraguá station (green diamond in Fig. 1b) located in the highest point of the MASP (1709 m.a.s.l.), reported higher O_3_ concentrations than the ones located inside the cities.

### Formulas of performance statistic models

Table S1 shows the formulas of the performance of statistic metrics used in our studies dataset.

### Table S1. Formulas of performance of statistic metrics

| Name | Formula |
| --- | --- |
| Mean Bias (MB) | $MB= \frac{1}{N}\sum(M_{i}- O_{i})$ |
| Mean absolute error (MAE) | $ME= \frac{1}{N}\sum\left\vert M_{i}- O_{i} \right\vert$ |
| Pearson correlation coefficient (R) | $R= \frac{\sum\left[ \left( M_{i}- \bar{M} \right)\left( O_{i}- \bar{O} \right) \right]}{\sqrt{\sum\left( M_{i}- \bar{M} \right)^{2}\sum\left( O_{i}- \bar{O} \right)^{2}}}$ |
| Root mean square error (RMSE) | $RMSE= \sqrt{\frac{1}{N}\sum{(M_{i}- O_{i})}^{2}}$ |
| Index of agreement (IOA) | $IOA=1- \frac{\sum\left( M_{i}- O_{i} \right)^{2}}{\sum\left( \left\vert M_{i}- \bar{O} \right\vert+\left\vert O_{i}- \bar{O} \right\vert\right)^{2}}$ |
| Normalize mean bias (NMB) | $NMB= \frac{\sum\left( M_{i}- O_{i} \right)}{\sum O_{i}}\times100$ |
| Normalize mean error (NME) | $NME= \frac{\sum\left\vert M_{i}- O_{i} \right\vert}{\sum O_{i}}\times100$ |
| Mean normalize bias (MNB) | $MNB=\frac{1}{N}\sum\frac{(M_{i}- O_{i})}{O_{i}}\times100$ |
| Mean normalize error (MNE) | $MNE=\frac{1}{N}\sum\frac{\vert M_{i}- O_{i}\vert}{O_{i}}\times100$ |
| Mean fractional bias (MFB) | $MFB= \frac{2}{N}\sum\frac{(M_{i}- O_{i})}{(M_{i}+ O_{i})}\times100$ |
| Mean fractional error (MFE) | $MFE= \frac{2}{N}\sum\frac{\vert M_{i}- O_{i}\vert}{(M_{i}+ O_{i})}\times100$ |
| Pair peak accuracy (PPA) | $PPA=\frac{(M_{i}- O_{max})}{O_{max}}\times100$ |
| Fraction of prediction within a factor of two (FAC2) | $FAC2=0.5\leq\frac{M_{i}}{O_{i}}\leq2.0$ |

M_i_ and O_i_ are paired in time model result and observations. $\bar{M}$ and $\bar{O}$ are the arithmetic mean of model results and observation, respectively.

### References

Andrade, M. de F., Kumar, P., de Freitas, E. D., Ynoue, R. Y., Martins, J., Martins, L. D., … Zhang, Y. (2017). Air quality in the megacity of São Paulo: Evolution over the last 30 years and future perspectives. *Atmospheric Environment*, *159*, 66–82. https://doi.org/10.1016/j.atmosenv.2017.03.051

McRae, G. J., Goodin, W. R., & Seinfeld, J. H. (1982). Development of a second-generation mathematical model for Urban air pollution-I. Model formulation. *Atmospheric Environment (1967)*, *16*(4), 679–696. https://doi.org/10.1016/0004-6981(82)90386-9

McRae, G. J., & Seinfeld, J. H. (1983). Development of a second-generation mathematical model for urban air pollution-II. Evaluation of model performance. *Atmospheric Environment (1967)*, *17*(3), 501–522. https://doi.org/10.1016/0004-6981(83)90124-5

Carter, W. P. L. (2000). *IMPLEMENTATION OF THE SAPRC-99 CHEMICAL MECHANISM INTO THE MODELS-3 FRAMEWORK Report to the United States Environmental Protection Agency*.

Byun, D., and Schere, K. L. (March 1, 2006). "Review of the Governing Equations, Computational Algorithms, and Other Components of the Models-3 Community Multiscale Air Quality (CMAQ) Modeling System." ASME. *Appl. Mech. Rev*. March 2006; 59(2): 51–77. <https://doi.org/10.1115/1.2128636>

Elbern, H., Strunk, A., Schmidt, H., & Talagrand, O. (2007). Emission rate and chemical state estimation by 4-dimensional variational inversion. In *Atmos. Chem. Phys* (Vol. 7). [www.atmos-chem-phys.net/7/3749/2007/](http://www.atmos-chem-phys.net/7/3749/2007/)

Grell, G. A., Peckham, S. E., Schmitz, R., McKeen, S. A., Frost, G., Skamarock, W. C., & Eder, B. (2005). Fully coupled “online” chemistry within the WRF model. *Atmospheric Environment*, *39*(37), 6957–6975. https://doi.org/10.1016/j.atmosenv.2005.04.027

Skamarock, W. C., Klemp, J. B., Dudhia, J., Gill, D. O., Zhiquan, L., Berner, J., Wang, W., Powers, J. G., Duda, M. G., Barker, D. M., & Huang, X.-Y. (2019). A Description of the Advanced Research WRF Model Version 4. *NCAR Technical Note NCAR/TN-475+STR*, 145. <https://doi.org/10.5065/1dfh-6p97>

Pielke, R. A., Cotton, W. R., Walko, R. L., Trembaek, C. J., Lyons, W. A., Grasso, L. D., Nieholls, M. E., Moran, M. D., Wesley, D. A., Lee, T. J., & Copeland, J. H. (1992). Meteorology and Atmospheric Physics A Comprehensive Meteorological Modeling System-RAMS. In *Meteorol. Atmos. Phys* (Vol. 49).

Freitas, E. D., Martins, L. D., Da Silva Dias, P. L., & De Fátima Andrade, M. (2005). A simple photochemical module implemented in RAMS for tropospheric ozone concentration forecast in the metropolitan area of São Paulo, Brazil: Coupling and validation. *Atmospheric Environment*, *39*(34), 6352–6361. <https://doi.org/10.1016/j.atmosenv.2005.07.017>

Freitas, S. R., Longo, K. M., Silva Dias, M. A. F., Chatfield, R., Dias, P. S., Artaxo, P., … Panetta, J. (2009). The coupled aerosol and tracer transport model to the Brazilian developments on the regional atmospheric modeling system (CATT-BRAMS)-Part 1: Model description and evaluation. *Atmospheric Chemistry and Physics*, *9*(8), 2843–2861. <https://doi.org/10.5194/acp-9-2843-2009>

Longo, K. M., Freitas, S. R., Pirre, M., Marécal, V., Rodrigues, L. F., Panetta, J., … Bela, M. (2013). The Chemistry CATT-BRAMS model (CCATT-BRAMS 4.5): A regional atmospheric model system for integrated air quality and weather forecasting and research. *Geoscientific Model Development*, *6*(5), 1389–1405. <https://doi.org/10.5194/gmd-6-1389-2013>

Riojas-Rodríguez, H., da Silva, A. S., Texcalac-Sangrador, J. L., & Moreno-Banda, G. L. (2016). Air pollution management and control in Latin America and the Caribbean: Implications for climate change. *Revista Panamericana de Salud Publica/Pan American Journal of Public Health*, *40*(3), 150–159.

CETESB. (2021). *Relatório de Qualidade do Ar no Estado de São Paulo-2020*. <http://cetesb.sp.gov.br/ar/publicacoes-relatorios/>
